# Role of PD-L1 Expression in Non-Small Cell Lung Cancer and Their Prognostic Significance according to Clinicopathological Factors and Diagnostic Markers

**DOI:** 10.3390/ijms20040824

**Published:** 2019-02-14

**Authors:** Konrad Pawelczyk, Aleksandra Piotrowska, Urszula Ciesielska, Karolina Jablonska, Natalia Glatzel-Plucinska, Jedrzej Grzegrzolka, Marzenna Podhorska-Okolow, Piotr Dziegiel, Katarzyna Nowinska

**Affiliations:** 1 Department of Thoracic Surgery, Wroclaw Medical University, Wroclaw 53-439, Poland; kopaw@wp.pl; 2 Department of Thoracic Surgery, Lower Silesian Centre of Lung Diseases, Wroclaw 53-439, Poland; 3 Department of Human Morphology and Embryology, Wroclaw Medical University, Wroclaw 50-368, Poland; aleksandra.piotrowska@umed.wroc.pl (A.P.); urszula.ciesielska@umed.wroc.pl (U.C.); karolina.jablonska@umed.wroc.pl (K.J.); n.m.glatzel@gmail.com (N.G.-P.); jedrzej.grzegrzolka@gmail.com (J.G.); marzenna.podhorska-okolow@umed.wroc.pl (M.P.-O.); piotr.dziegiel@umed.wroc.pl (P.D.); 4 Department of Physiotherapy, Wroclaw University School of Physical Education, Wroclaw 51-612, Poland

**Keywords:** PD-L1, Ki-67, TTF-1, p63, non-small cell lung cancer

## Abstract

Background: The latest immunotherapy, used in the treatment of non-small cell lung cancer (NSCLC), uses monoclonal antibodies directed against programmed death ligand 1 (PD-L1) to inhibit its interaction with the PD-1 receptor. Elevated levels of PD-L1 expression were observed on NSCLC cells. The association between PD-L1 expression and clinicopathological features is still unclear. Therefore, we examined this relationship and also compare PD-L1 expression levels with Ki-67, p63 and TTF-1. Methods: 866 samples of NSCLCs were used to prepare tissue microarrays (TMAs) on which immunohistochemical (IHC) reactions were performed. Changes in the level of *CD274* (*PD-L1*) gene expression in 62 NSCLC tumors were tested in relation to 14 normal lung tissues by real-time PCR reactions (RT-PCR). Results: PD-L1 expression was observed in 32.6% of NSCLCs. PD-L1 expression was increased in higher malignancy grades (G) (*p* < 0.0001) and in higher lymph node status (pN) (*p* = 0.0428). The patients with low PD-L1 expression had longer overall survival compared to the group with high expression (*p* = 0.0332) in adenocarcinoma (AC) only. Conclusions: PD-L1 expression seems to be associated with increased tumor proliferation and aggressiveness as well as shorter patient survival in NSCLC, predominantly in the AC group.

## 1. Introduction

Lung cancer is one of the main causes of cancer-related deaths worldwide. According to the American Cancer Society, the number of deaths from lung cancer is higher than the number of deaths caused by breast, colon and prostate cancers [1]. Non-small cell lung cancer (NSCLC) constitutes over 80% of all lung cancer cases [2]. This group includes mainly squamous cell carcinoma (SCC) and adenocarcinoma (AC) [3]. Early diagnosis and the use of the latest targeted therapies have contributed to a slight improvement in prognosis in patients even in advanced stages of the disease [2]. Immunotherapy, which is a novel method of lung cancer treatment, also contributes to such improvements. The latest method of immunotherapy uses monoclonal antibodies directed against the immune-checkpoint molecules such as receptor programmed cell death 1 (PD-1) or its ligand (PD-L1). Their roles in the first- or second-line treatment of advanced stages of NSCLC are well established, but the association between PD-L1 expression and clinicopathological features is still unclear and discussed in the literature [4]. 

Programmed death ligand 1 (PD-L1), also known as CD274, is considered an immune checkpoint facilitating anti-tumor suppression of the immune pathway [5]. PD-L1 can be expressed on the surface of various cells. The main inducer of PD-L1 expression in vivo is interferon gamma (IFN-γ), which is released by CD8^+^ T-cells [4]. The expression of PD-L1 is observed on the surface of macrophages, antigen-presenting cells, B and T lymphocytes, epithelial, muscle and endothelial cells [5], whereas PD-1 receptor is expressed predominantly by activated cytotoxic T cells. PD-L1 ligand binds to PD-1 receptor on activated T cells and this connection results in suppression of the immune system [5,6]. The connection of PD-1 with PD-L1 prevents an autoimmune response in peripheral tissues during inflammation [7]. The ligand–receptor complex elicits two reactions which inhibit the immune response. The first effect is inhibition of interleukin 2 (IL-2) synthesis [8]. Another additional effect of PD-L1 engagement is related to the inhibition of the T cell receptor, known as a “stop signal”. This pathway can alter the duration of T cell contact with target cells and antigen-presenting cells [9]. 

Elevated levels of PD-L1 expression were also observed on the cell surface of different types of cancer cells, including NSCLC. It is believed that PD-L1 expression allows cancer cells to avoid the immune response [6]. Monoclonal antibodies are currently successfully used to inhibit the interaction of the PD-1 receptor with the PD-L1 protein. These monoclonal antibodies bind to PD-1 receptors and make it impossible for PD-L1 to bind to the receptor. When cancer cells are unable to affect activated T cells, immune response remains active. Preclinical and clinical data indicated that monoclonal antibodies could significantly enhance patient antitumor immune responses [9]. The identification and characterization of factors to determine patients with good response to immunotherapy seems to be very important, particularly in the case of two main histopathological subgroups of NSCLC i.e., AC and SCC [10]. Additionally, the association of PD-L1 expression with clinicopathological factors is still unclear. For this reason, we analyzed the relationship between PD-L1 expression and clinicopathological factors to determine its prognostic and predictive value on a large and homogenous group of patients. To the best of our knowledge, only a few studies have analyzed the level of PD-L1 expression in NSCLC on such a large group of patients as ours. Another aim of the study was to determine the level of expression and localization of PD-L1 ligand in NSCLC and to compare it with the expression level of commonly used diagnostic markers such as Ki-67 proliferation antigen, p63 and thyroid transcription factor 1 (TTF-1) proteins, which are routinely used to distinguish a morphological subtype of NSCLC.

## 2. Results

### 2.1. Comparison of Programmed Death Ligand 1 (PD-L1) Expressions in Non-Small Cell Lung Cancer (NSCLC) Subtypes

**Figure 1:**
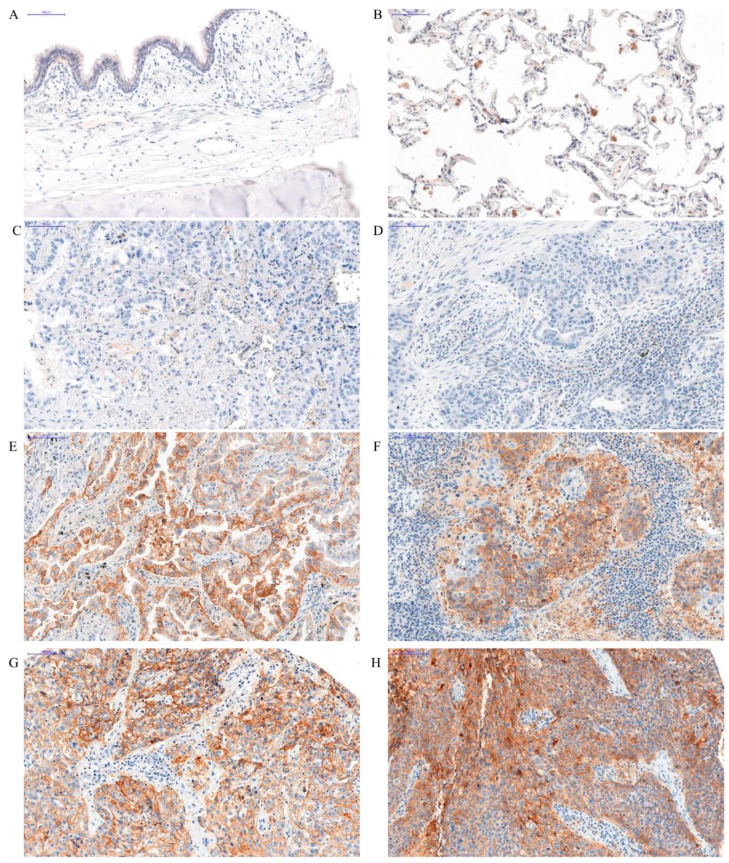
Positive membranous immunohistochemical reaction (brown) indicating PD-L1 expression performed on healthy lung tissue (**A**,**B**) and in different grades of malignancy in adenocarcinoma (AC) (**C**,**E**,**G**) and squamous cell cancer (SCC) (**D**,**F**,**H**). Lack of PD-L1 expression—healthy lung tissue (**A**) and PD-L1 expression in macrophages—positive control (**B**). Expression of PD-L1 increased in higher malignancy grade in AC—G1 (**C**), G2 (**E**), and G3 (**G**), and in SCC—G1 (**D**), G2 (**F**) and G3 (**H**), magnification, ×200.

**Figure 2:**
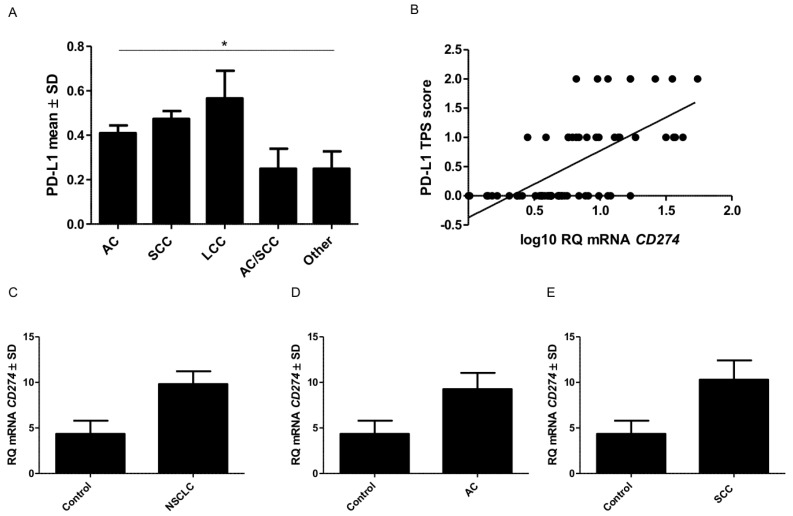
PD-L1 protein expression and *CD274* mRNA in non-small cell lung cancer (NSCLC) are higher than in healthy lung tissues. Comparison of PD-L1 protein expression levels detected by immunohistochemistry (IHC) (**A**) in different subtypes of NSCLC (* *p* = 0.0074). Positive correlation between mRNA expression levels of *CD274* detected by the real-time PCR and PD-L1 expression detected by IHC evaluated by Tumor Proportion Score (TPS (0–2) (**B**). Comparison between expression of mRNA CD274 in healthy lung tissues and NSCLC (**C**) adenocarcinoma (AC) (**D**) squamous cell cancer (SCC) (**E**).

In the case of PD-L1, the membrane expression was found in cancer cells. PD-L1 expression was not observed in the normal pulmonary parenchyma (Figure 1A) but was observed in macrophages. The expression of PD-L1 in macrophages and lymphocytes is typical and was also observed in other studies [5]. The mean level of PD-L1 expression in NSCLC subtypes was significantly different (Kruskal-Wallis, *p* = 0.0206), but not between AC and SCC (U-Mann-Whitney, *p* = 0.0780) (Figure 2). The highest level of expression was noticed in large-cell lung carcinoma LCC (mean value 0.57 ± 0.12). In the AC subtype, the expression level was 0.41 ± 0.03, and 0.47 ± 0.03 in SCC. The percentage of patients with high or low PD-L1 expression in each group is shown in Table 1. Analysis with Dunn’s Multiple Comparison test indicated that there was no difference between PD-L1 protein expression levels in these two subtypes. We also evaluated the levels of mRNA expression of *CD274* gene which encodes PD-L1 protein. The obtained results showed that a higher level of mRNA was found in NSCLC compared to the control group (Figure 2). A similar relationship was also observed in AC and SCC. This difference was not statistically significant. We compared the level of *CD274* mRNA and PD-L1 protein expression. A graph showing the positive strong correlation between them (Spearman r = 0.67; *p* < 0.0001) is shown in Figure 2B. 

### 2.2. Comparison of PD-L1 with TTF-1, p63 and Ki-67 Antigen Expression Levels

Ki-67, p63 and TTF-1 markers revealed nuclear expression in cancer cells. We observed a low positive correlation between PD-L1 vs. Ki-67 (r = 0.16, *p* < 0.0001) and p63 (r = 0.10, *p* = 0.0065) in NSCLC. Similar results were also observed in the AC subtype. We observed low correlation between PD-L1 vs. Ki-67 (r = 0.18, *p* = 0.0007) and p63 (r = 0.16, *p* = 0.0013). In the SCC subtype, PD-L1 revealed a low positive correlation with Ki-67 (r = 0.12, *p* = 0.0098) and TTF-1 (r = 0.11, *p* = 0.0191).

### 2.3. The Associations between PD-L1 Expression and Clinicopathological Parameters

**Figure 3:**
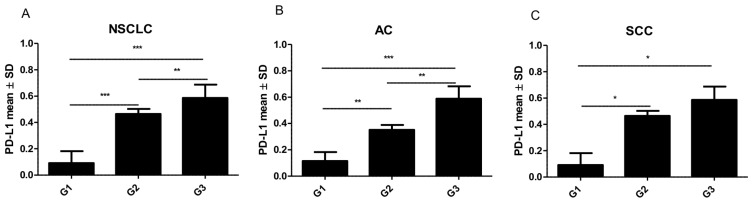
The higher the grade (G) of malignancy, the higher the PD-L1 expression observed. Comparison of PD-L1 protein expression levels in different grades of malignancy (G) of non-small cell lung cancer (NSCLC) (**A**), adenocarcinoma (AC) (**B**), squamous cell cancer (SCC) (**C**) * *p* ≤ 0.05, ** *p* ≤ 0.01, *** *p* ≤ 0.001.

PD-L1 expression in NSCLC cells was compared with clinicopathological factors. Due to the fact that AC and SCC are the main groups of NSCLC, we analyzed and described the relation between PD-L1 expression and clinicopathological factors in both groups of NSCLC and according to histological subtypes. The higher grade (G) of malignancy, the higher PD-L1 expression was observed (Kruskal-Wallis test, *p* < 0.0001) (Figure 1). Similarly, in the group of AC, there was also a noticeable difference in the level of PD-L1 expression i.e. the higher the grade (G) of malignancy, the higher the increase in PD-L1 reported (Kruskal-Wallis test, *p* = 0.0004) (Figure 1B–D), unlike SCC (Kruskal Wallis test, *p* = 0.0937) in which PD-L1 expression was also increased in higher grades, but the differences were statistically significant only between G1 vs. G2 and G1 vs. G3 (Figure 1F–H, 3). The comparison of PD-L1 expression levels in G1 vs. G2, G1 vs. G3 and G2 vs. G3 in AC and SCC is presented in Figure 3.

Increased PD-L1 expression was observed in relation to lymph node metastasis (Kruskal-Wallis test, *p* = 0.0428). The highest mean of expression was observed in cases of metastases to hilar and intrapulmonary lymph nodes (N1) in NSCLC and AC and SCC subtypes separately (Table 2). PD-L1 expression was higher in the N1 group compared to a group of patients without lymph node metastases (N0). A statistically significant difference was observed between these groups (N0 vs. N1). A similar tendency was observed in the AC subtype. The mean level of PD-L1 expression in tumors with mediastinal lymph node metastasis (N2) was higher than in the N0 group. However, the difference was statistically significant only in the AC subtypes. PD-L1 expression levels did not change depending on pathological T descriptor in NSCLC. The difference was significant between T1 vs. T2 and T1 vs. T3–T4 (Table 2).

However, PD-L1 expression was increased in higher stages of NSCLC. The difference between the expression in I vs. II stage and I vs. III–IV was close to statistical significance (Table 2) in NSCLC and AC subtype. Slightly higher levels of expression were observed with higher stages in the group of patients with SCC. 

We also examined the expression of PD-L1 proteins depending on the extent of tumor necrosis. Therefore, NSCLC cases were divided into groups depending on the percentage of necrosis: 0%–30%, 30%–70% and above 70%. PD-L1 expression was significantly different between the groups only in the AC subtype (Kruskal-Wallis, *p* = 0432). The difference between PD-L1 expression in the group with 0%–30% necrosis versus the group with 30%–70% necrosis was also significant (*p* = 0.0072).

### 2.4. The Associations between PD-L1 Expression and Overall Survival (OS)

**Figure 4:**
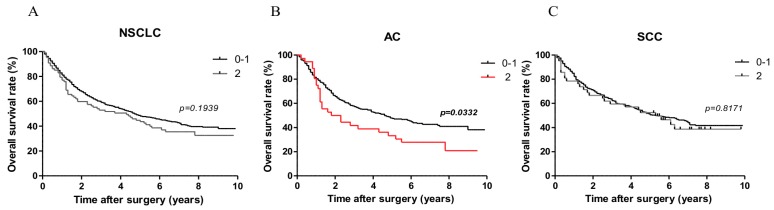
Patients with low PD-L1 expression (0–1 point) had longer survival compared to the group with high expression (2 points) (*p* = 0.0229) in AC subtype. Comparison of Kaplan-Meier curves presenting overall survival percentage in patients with NSCLC (**A**), AC (**B**), SCC (**C**) according to low (0–1) and high (2) levels of PD-L1 expression.

Log-rank (Mantel-Cox) analysis showed that in the AC group higher expression of PD-L1 (2 points) was related to overall survival (OS). Patients with low PD-L1 expression (0–1 point) had longer survival compared to the group with high expression (2 points) (*p* = 0.0332). However, such a difference was not observed when patients were divided into two groups i.e. one with PD-L1 expression (1–2 points) and one without expression (0 points). Moreover, we did not observe prognostic associations of PD-L1 expression with OS in patients with SCC. Patient survival curves are shown in Figure 4. The results of univariate and multivariate analysis are presented in Table 3. In the multivariate analysis in the AC subtype age ≥ 60 years, male sex, smoking history and advanced tumor size (pT) and lymph node status (pN) status were related to shorter OS. However, only advanced pT status was related to shorter OS in SCC.

## 3. Discussion

Our results indicate that PD-L1 expression detected by immunohistochemistry (IHC) was higher in NSCLC tissue compared to normal bronchial epithelium. RT-PCR reactions also showed higher PD-L1 expression in tumor tissues compared to normal lung parenchyma. This difference, however, was not statistically significant and it could have resulted from a small sample size, but we observed positive strong correlations between IHC and RT-PCR results of expression. Kim et al. [11] observed similar results when compared IHC PD-L1 expression and mRNA detected by RNA in situ hybridization. In our cohort, positive IHC PD-L1 expression (≥1%) was observed in 32.5% of NSCLC patients. High expression in ≥50% of tumor cells occurred in 10.6% of NSCLCs. The occurrence of positive PD-L1 expression was slightly lower than in other studies. Positive PD-L1 expression in the NSCLC group was most frequently observed between 50% and 70% of cases [12,13,14,15,16,17,18,19]. However, in a few studies PD-L1 positive cancer cells were found in a much smaller percentage [20,21,22,23]. Cooper et al. [20] conducted a study on a group of 678 patients. Their cohort was slightly smaller than ours and IHC was performed using tissue microarrays (TMAs). Those researchers observed membranous PD-L1 expression of any intensity in only 32.8% cases. High expression of PD-L1 was revealed in 7.4% of NSCLC. These data are comparable to the results obtained in our study. 

PD-L1 protein expression differs significantly depending on clinical studies on NSCLC. Additionally, data on the relationship between PD-L1 expression and clinicopathological factors are very different. Most studies in which high PD-L1 expression (50%–72.7%) was reported were conducted in an Asian population [12]. In our cohort, involving a Western population, the incidence was lower, which is similar to other studies on this ethnic group [12,16,24]

Another explanation for diverse expression of PD-L1 in NSCLC may be related to the use of different scoring methods and cut-off levels for evaluation. Chen et al. [15] detected PD-L1 expression in 65.3% of NSCLC cases using the IRS scale (according to Remmele and Stegner [25]; a product of percentage of stained cells (0–4) and staining intensity (0–3)). Positive IHC for PD-L1 was observed when the cut-off value was above 3. However, in the study of Cooper et al. [20] in which PD-L1 expression was noted in 32% of patients, positive expression was found when over 50% of cells were PD-L1 positive. In that study, the intensity of IHC was not considered whereas Tang et al. considered IHC as positive when PD-L1 expression was observed in more than 5% of NSCLC cells. In their study, the percentage of patients with a positive reaction was 65.9%, which was much higher than in the study of Cooper et al. [20]. In our study, we used the tumor proportion score (TPS), which is currently routinely used in diagnostics. Response to immunotherapy is assessed by the TPS score (<1%, ≥1% to 49%, ≥50%) [4,26,27,28]. The differences in the percentage of patients expressing PD-L1 in NSCLC cells in various studies could probably result from the use of different antibody clones (22C3, 22-8, SP142 and SP263). These antibodies were validated in clinical trials for various PD-1/PD-L1 inhibitors. Therefore, various monoclonal anti-PD-L1 antibodies were also used in other studies. For this reason, the obtained results are difficult to compare [12]. In our study we used the same 22C3 antibody as in the study of Cooper et al. We also used TMAs and similar cut-off levels as the above researchers. These three aspects could be the reason why similar results of positive PD-L1 expression in NSCLC were obtained in both studies [12,20].

According to recent studies, in terms of the evaluation of PD-L1 expression in NSCLC cells, too small biopsy specimens (e.g., cell-blocks or core needle biopsy) may not be representative of the whole tumor issue [11,29]. This is mostly due to heterogeneity and dynamic dispersion of PD-L1 expression in the tumor tissue. Evaluation of small specimens and different sites of necrosis in the tumor could be the reasons for diverse expression of PD-L1 in NSCLC. In our study we found a significantly higher expression of PD-L1 in cancer tissue affected by necrosis (>70%), particularly in the AC group. According to the TPS, necrotic areas should be excluded from evaluation. Increased PD-L1 expression is frequently observed in the necrotic area and it could be one of the reasons leading to variable study results.

Additionally, we observed an association of PD-L1 expression with gender and smoking status. The male sex and smoking were related to shorter survival onlyin the AC group. Li et al. [30] conducted a meta-analysis in 1981 of patients with NSCLC. The study compared the response to treatment with PD-1 inhibitors and chemotherapy according to smoking status. Li et al. indicated that inhibitors of PD-1 were more effective in NSCLC patients with smoking history compared to chemotherapy. The explanation of this could be that smoking will impact the anti-tumor response by altering the number of Treg cells and the function of natural killer cells [30].

In our study, we also examined the association of the PD-L1 protein with the prognostic and diagnostic markers, i.e., Ki-67 antigen, TTF-1 factor and p63 proteins. We observed weakly positive correlations of PD-L1 with all the markers. Of note, the highest correlations of PD-L1 with Ki-67 were observed for the AC subtype. Ki-67 antigen is routinely used as a marker to determine the proliferative potential of cancer cells. Its expression is observed in the cell nucleus immediately before or during mitosis and is associated with chromatin condensation and the separation of sister chromatids [31,32,33]. The correlation between PD-L1 expression and Ki-67 antigen expression shows the association of PD-L1 expression with tumor cell proliferation. This is also confirmed by the increase in PD-L1 expression observed in higher grades of malignancy. Igarashi et al. [29] also observed higher expression of PD-L1 in G2 and G3 in AC compared to G1 in AC. In their meta-analysis, Zhang et al. [34] also reported higher PD-L1 expression in the case of the higher grade. Similar to our study, Takada et al. [35] presented a positive mean correlation of the expression level of Ki-67 antigen and PD-L1 protein. Their study included 205 patients with SCC. In our study, the correlation was weak. However, our study included a much larger group (i.e., 866 patients with NSCLC). In addition, higher Spearman’s correlation coefficient was observed for the AC subtype than for SCC. Escape of cancer cells from the immune system due to PD-L1 expression explains the effect of this protein on increased tumor cell proliferation [10]. This process should lead to tumor growth and should contribute to worse prognosis of patients with high PD-L1. A larger tumor size and a lower grade of tumor-infiltrating lymphocytes (TILs) is often observed in patients with high PD-L1 expression [36]. Parea et al. [36] examined the expression of PD-L1 and human leukocyte antigens-1 (HLA-I) in NSCLC and its association with TILs. They observed that the co-expression pattern of both markers is strongly associated with TILs, as well as tumor grade and stage. In patients with the PD-L1+/HLA-I pattern, tumors were larger (T3 + T4) and had a lower grade of TILs. Parea et al., however, did not observe such associations with the stage or grade when they examined PD-L1 or HLA-1 expression separately. This may explain why we did not observe higher PD-L1 expression in larger-sized tumors. Researchers suggest that in addition to PD-L1 expression, the presence of HLA-1 may also be important to predict good patient response to immunotherapy. Further studies are warranted to confirm this hypothesis [36].

We also observed a positive correlation between the expression of PD-L1 and p63 which belongs to the same family of transcription factors as p53 and which is known for its role in epidermal development. p63 is overexpressed in SCC [37]. Therefore, p63 protein is a marker used to differentiate SCC from other NSCLC subtypes. The association of PD-L1 expression with p63 could be related to PD-L1 overexpression in the SCC subtype. High expression of PD-L1 in SCC was also observed by other authors [4,6,12,38,39]. The relationship between the expression of these two proteins has not been studied yet. However, Shimoji et al. [6] observed that the presence of PD-L1 expression correlates with a high expression of p53.

In addition, Shimoji et al. [6] did not report a relationship between PD-L1 expression and clinicopathological factors in the SCC subtype, unlike in the case of the AC subtype. This is consistent with our results. We did not observe a relationship between PD-L1 expression and tumor stage, TNM characteristics or patient survival in the SCC subtype. However, in the AC subtype, PD-L1 expression was associated with the grade (G) of malignancy, lymph node metastases and patient survival. Our study showed that AC patients with high expression (≥50%, 2 point) of PD-L1 had shorter survival times. Shimoji et al. observed exactly the same relationship for the AC subtype. This relationship was not observed in the SCC subtype. Other authors revealed significantly worse prognosis in AC patients when PD-L1 expression exceed 1% (≥1%) which was not only related to OS but also progression-free survival (PFS) [6,38,39]. However, Cooper et al. [20] observed a different relationship. In their study, patients with higher PD-L1 expression (≥50%) were characterized by longer survival in the SCC subtype. This relationship was not observed in patients with AC. However, some authors emphasized significantly shorter survival in the SCC group [40]. However, most authors considered NSCLC patients from three meta-analyses [16,20,38,41,42,43]. Zhong et al. [44] in their meta-analysis based on 12 studies involving 1653 patients indicated no statistically significant difference between PD-L1 expression and prognosis in NSCLC, which is similar to our study results. To avoid these discrepancies the analyses should be performed on subgroups of NSCLC with consideration given to histology and ethnicity as in the study by Zhong et al. [44] and a similar definition of positive PD-L1 expression. Our study included a larger number of patients of the Western population. It was based on recommended TPS cut-off values and the results indicated significantly worse prognosis only in patients with high PD-L1 expression (2 points) in the AC group. According to our results in the AC group, identification of PD-L1 expression level could be more important and useful than in other NSCLCs. Due to these conflicting results, further research is warranted to determine the influence of PD-L1 expression on patient survival and its role as a prognostic marker.

Our study, similar to studies based on retrospectively collected data, is not free from several limitations. Although it was conducted on one of the largest cohorts, it is a single institutional study and the results still need to be validated in a multicenter group of patients. Also, TMAs, which were used in our study, have some limitations. Tissue microarrays were prepared from small tissue sections. However, we selected representative and evenly distributed areas of NSCLC of all the available tissue. The TMA method is considered the best technique to evaluate such a large study group.

## 4. Materials and Methods

### 4.1. Patient Cohort

From January 2007 to December 2011, 1371 consecutive patients diagnosed with lung cancer underwent surgery (curative resection) at the Department of Thoracic Surgery of Wroclaw Medical University. All patients underwent major resection of lung parenchyma (pneumonectomy, lobectomy) or sublobar resection (segmentectomy, wedge resection) due to impaired lung function. Archival and frozen material of lung cancer tissue specimens was obtained from 892 patients. Eleven patients were excluded from this group due to prior chemotherapy, six patients due to small cell lung cancer and nine due to typical carcinoid tumor. The data obtained from the remaining 866 patients with NSCLC underwent further retrospective analysis. Histopathological reports with clinical data from the Polish National Registry of Lung Cancer were evaluated. All patients gave their written informed consent. The study concept was approved by Wroclaw Medical University institutional review board and bioethics committee (ID No. KB-83/2011;03.03.2011).

Histopathological evaluation and pathological staging was performed according the criteria of the World Health Organization (WHO, 2015). The archival material consisted of 866 cases of NSCLC tumors, including ACs (*n* = 364), SCCs (*n* = 381), adenosquamous carcinomas (*n* = 32), large cell carcinomas (LCCs) (*n* = 31) and other and unclassified NSCLC (*n* = 58). Patients characteristics are presented in Table 1. The control group consisted of 140 healthy lung tissue sections.

### 4.2. Tissue Microarrays (TMAs)

Thirty-seven tissue microarrays (TMAs) were prepared from 866 paraffin blocks with sections of NSCLC. As a control we used four TMAs with 140 sections of normal lung tissue from resected lung parenchyma.

For this purpose, histological slides stained with hematoxylin and eosin were obtained from archival material. The slides were scanned using the Pannoramic Midi II histological scanner (3DHISTECH Ltd, Budapest, Hungary). Using the Pannoramic Viewer Program (3DHISTECH Ltd.), 3 representative cancer sites were selected, followed by the transfer with a core size of 1.5 mm to the tissue arrays using the TMA Grand Master (3DHISTECH Ltd.).

### 4.3. Immunohistochemical Reaction (IHC)

Each TMA was sectioned at 4 μm and IHC reactions were performed using primary antibodies detecting the expression of the tested proteins. Deparaffinisation, hydration and thermal demasking of epitopes were performed using Dako PT Link (Dako, Glostrup, Denmark). The slides were incubated for 30 minutes at 97 °C (low pH Target retrieval solution; Agilent Technologies, Santa Clara, CA, USA). Monoclonal mouse anti-PD-L1 antibody (1:50 dilution; clone 22C3 (concentrate); code No. M3653; Dako) was used to detect the ligand using enhancer signal EnVision ™ FLEX + Mouse LINKER (Dako). Detection of other markers was performed using monoclonal mouse anti-Ki-67 antibody (ready to use, Clone MIB-1, code IS626; Dako), anti-TTF-1 (ready to use, Clone 8G7G3/1, code IR056, Dako) and anti-p63 (ready-to-use, Clone DAK-p63, code IR662, Dako). The immunohistochemical reactions were performed in an automatic system DAKO Autostainer Link48 (Dako). The EnVision FLEX kit (Dako) was used to visualize the antigens and the preparations were additionally stained with Mayer’s hematoxylin. To differentiate between AC and SCC, IHC with TTF-1 and p63 was performed.

### 4.4. Evaluation of IHC Reaction

Two independent pathologists conducted the assessment. The estimation of membranous PD-L1 and nuclear TTF-1, p63 and Ki-67 antigen expression was done at magnification of ×200 with the use of BX41 (Olympus, Tokyo, Japan) light microscope coupled with visual circuit and CellD (Olympus) software for computer image analysis. The intensity of TTF-1, p63 and Ki-67 expression was determined with the use of a five-point scale (0—no expression, 1 point—1%–10%, 2 points—11%–25%, 3 points—26%–50%, 4 points >50%) [45]. The estimation of PD-L1 expression was performed using the Tumor Proportion Score (TPS) that is applied routinely in diagnostic settings [4,27,28]. It is a three-point evaluation scale (0 point <1%, 1 point ≥1% to 49% and 2 points ≥50%). Cytoplasm staining in tumor cells was not considered.

### 4.5. Real-Time PCR (RT-PCR)

Frozen material of 62 sections of NSCLC and 14 control sections of normal lung tissue were used for RT-PCR. RNeasy Mini Kit was used (Qiagen, Hilden, Germany) for RNA isolation. The reverse transcription reaction was performed using High-Capacity cDNA Reverse Transcription Kit with RNase Inhibitor (Applied Biosystems, Waltham, MA, USA). Changes in the expression level of PD-L1 (*CD274*; TaqMan Gene Expression Assay, Applied Biosystems) were tested using 7900HT Fast Real-Time PCR System (Applied Biosystems). Relative quantification (RQ) method was applied. The analysis of *CD274* gene expression was performed using the RQ Manager 1.2 software (Applied Biosystems). The results were standardized, based on the expression of the reference gene of β-actin (*ACTB*; TaqMan Gene Expression Assay, Applied Biosystems). Changes in the level of *CD274* gene expression in NSCLC cells were assessed in relation to normal lung cells. The evaluation of *CD274* gene expression by real-time PCR was repeated three times. The obtained results were shown in the graphs on a logarithmic scale and subjected to statistical analysis.

### 4.6. Statistical Analysis

Spearman’s rank correlation was used to evaluate the relationship between the expression of p63 and TTF-1 proteins as well as Ki-67 antigen. Kruskal-Wallis with Dunn’s Multiple Comparison test was used to assess the association between the intensity of PD-L1 protein expression and NSCLC subtypes. Mann-Whitney U and Chi2 tests were used to assess the clinicopathological factors of the examined NSCLC samples. Overall survival (OS) was measured from the date of surgery to the date of death or the last follow-up. The Kaplan-Meier analysis and log-rank test were used to verify the relationship between the intensity of PD-L1 expression and patient survival. Cox proportional hazard regression model was used to evaluate the clinicopathological characteristics related to OS (hazard ratio—HRs and 95% confidence intervals—CIs). The statistical significance of the differences in mRNA expression of CD274 gene in normal lung tissues and NSCLC was determined using Kruskal-Wallis test. The results were considered statistically significant if two-sided *p* values were ≤0.05. Statistical analysis was performed using Prism 5.0 (GraphPad, La Jolla, CA, USA) and Statistica 13.1 (Dell, Round Rock, TX, USA).

## 5. Conclusions

PD-L1 expression seems to be associated with increased tumor proliferation and aggressiveness as well as shorter patient survival in the AC group. However, the relationship between the expression level of PD-L1 protein and clinicopathological factors is not clear and studies often reveal contradictory results. Further research is warranted to determine the relationship between PD-L1 expression and the clinicopathological factors of NSCLC.

## Figures and Tables

**Table 1:** Clinicopathological characteristics of non-small cell lung cancer (NSCLC) patients related to programmed death ligand 1 (PD-L1) expression. Percentages in brackets are relative to the total of 866 cases.

Clinicopathological Parameter	NSCLC				
	n 866 (%)	PD-L1 Expression			
		0	1	2	*Chi*^2^*test* *p* Value
		<1%	1–49%	≥50%	
		584 (67.4%)	193 (22.3%)	89 (10.3%)	
***Age ≤ 60***	357 (41.2%)	240 (27.7%)	78 (9%)	39 (4.5%)	1.000
***> 60***	509 (58.8%)	344 (39.7%)	115 (13.3%)	50 (5.8%)	
***Sex Male***	640 (73.9%)	428 (49.4%)	145 (16.7%)	67 (7.73%)	**<0.0001**
***Female***	226 (26.1%)	155 (17.9%)	48 (5.5%)	178 (20.6%)	
***Smoking status*** ***Yes***	722 (83.4%)	483 (55.8%)	157 (18.1%)	82 (9.5%)	**0.0107**
***No***	126 (14.5%)	101 (11.7%)	18 (2.1%)	7 (0.8%)	
***Histology type AC***	364 (42%)	252 (29.1%)	74 (8.5%)	38 (4.4%)	0.0522
***SCC***	381 (44.0%)	244 (28.2%)	95 (11.0%)	43 (5.0%)	
***AC and SCC***	32 (3.7%)	25 (2.9%)	6 (0.7%)	1 (0.1%)	
***Other and Unclassified carcinomas***	57 (6.6%)	46 (5.3%)	6 (0.7%)	5 (0.6%)	
***LCC***	31 (3.6%)	16 (1.8%)	12 (1.4%)	3 (0.3%)	
***Tumor size (T) T1***	216 (24.9%)	145 (16.7%)	49 (5.7%)	22 (2.5%)	0.9951
***T2***	374 (43.2%)	248 (28.6%)	89 (10.3%)	37 (4.3%)	
***T3***	182 (21%)	121 (14.0%)	42 (4.8%)	19 (2.2%)	
***T4***	94 (10.9%)	70 (8.1 %)	13 (1.5%)	11 (1.3%)	
***Lymph nodes (N) N0***	574 (66.3%)	403 (46.5%)	116 (13.4%)	55 (6.4%)	0.5176
***N1***	151 (17.4%)	89 (10.3%)	46 (5.3%)	16 (1.8%)	
***N2–3***	141 (16.3%)	92 (10.6%)	31 (3.6%)	18 (2.1%)	
***Metastasis (M) M0***	858 (99.0%)	578 (66.7%)	191 (22.1%)	89 (10.3%)	0.9987
***M1b***	8 (0.9%)	6 (0.7%)	2 (0.2%)	0 (0%)	
***Stage I***	315 (36.4%)	224 (25.9%)	67 (7.7%)	24 (2.8%)	0.8046
***II***	289 (33.4%)	187 (21.6%)	71 (8.2%)	31 (3.6%)	
***III–IV***	262 (30.3%)	173 (20.0%)	55 (6.4%)	34 (3.9%)	
***Grade of malignancy (G) G1***	52 (6.0 %)	47 (5.4%)	4 (0.5%)	1 (0.1%)	**0.0114**
***G2***	667 (77.0%)	456 (52.6%)	148 (17.1%)	64 (7.3%)	
***G3***	147 (17.0%)	83 (9.6%)	41 (4.7%)	24 (2.7%)	

Abbreviations: NSCLC—non-small cell lung cancer, LCC—large-cell lung carcinoma, AC—adenocarcinoma, SCC—squamous cell carcinoma; significance in bold.

**Table 2:** Associations of PD- L1 expression level with clinicopathological characteristics in patients with NSCLC.

*Clinicopathological Parameters*	NSCLC	AC	SCC
***P* value (U-Mann–Whitney Test)**			
**Lymph nodes**			
**N0 vs. N1**	**0.0079**	**0.0143**	0.0917
**N0 vs. N2** **N1 vs. N2**	0.1088 0.2081	**0.0347** 0.3080	0.4296 0.1496
**Tumor size**			
**T1 vs. T2** **T1 vs. T3–4**	0.4382 0.3519	0.3644 0.2667	0.4032 0.4995
**T2 vs. T3–4**	0.2735	0.3638	0.3984
**Stage**			
**I vs. II** **I vs. III–IV** **II vs. III–IV**	0.0730 0.0800 0.9259	0.0628 0.0651 0.9507	0.5288 0.3834 0.7438
**Mean Value ± SD**			
**Lymph nodes**			
**N0**	0.39 ± 0.65	0.35 ± 0.63	0.45 ± 0.69
**N1** **N2**	0.51 ± 0.68 0.47 ± 0.71	0.56 ± 0.73 0.50 ± 0.73	0.53 ± 0.66 0.44 ± 0.71
**Tumor size**			
**T1** **T2**	0.43 ± 0.67 0.43 ± 0.66	0.44 ± 0.69 0.41 ± 0.68	0.47 ± 0.68 0.45 ± 0.66
**T3–4**	0.41 ± 0.67	0.37 ± 0.63	0.50 ± 0.73
**Stage**			
**I** **II** **III–IV**	0.36 ± 0.62 0.46 ± 0.68 0.46 ± 0.71	0.31 ± 0.59 0.46 ± 0.70 0.46 ± 0.72	0.42 ± 0.65 0.47 ± 0.67 0.52 ± 0.75

Abbreviations: NSCLC—non-small cell lung cancer, AC—lung adenocarcinoma, SCC—squamous cell lung cancer; significance in bold.

**Table 3:** Univariate and multivariate Cox proportional hazards analysis in 866 patients with NSCLC (344 AC, 381 SCC).

Clinicopathological Parameter	NSCLC		AC		SCC	
	Univariate analysis HR (95% CI) *p*	Multivariate analysis HR (95% CI) *p*	Univariate analysis HR (95% CI) *p*	Multivariate analysis HR (95% CI) *p*	Univariate analysis HR (95% CI) *p*	Multivariate analysis HR (95% CI) *p*
**Age** **≤60 vs. >60**	1.33 (1.12–1.61) **0.0017**	1.41 (1.16–1.70) 0.0003	1.40 (1.07–1.84) **0.0150**	1.68 (1.27–2.22) **0.0002**	1.22 (0.90–1.63) 0.1937	
**Sex** **Male vs. Female**	1.15 (0.94–1.40) 0.1823		1.82 (1.35–2.44) **<0.0001**	1.70 (1.27–2.22) **0.0007**	1.15 (0.80–1.62) 0.4502	
**Smoking history** **yes vs. no**	1.27 (0.97–1.63) 0.0683		1.50 (1.02–2.21) **0.0387**	1.43 (0.95–2.13) 0.0762	1.87 (1.41–2.48) **<0.0001**	1.25 (0.78–2.01) 0.3602
**pT** **T1–T2 vs. T3–T4**	1.86 (1.55–2.22) **<0.0001**	1.56 (1.25–1.92) **<0.0001**	2.04 (1.54–2.71) **<0.0001**	1.60 (1.14–2.24) **0.0052**	1.75 (1.33–2.32) **<0.0001**	1.55 (1.11–2.16) **0.0102**
**pN** **N0 vs. N+**	1.83 (1.53–2.18) **<0.0001**	1.50 (1.20–1.87) **0.0004**	2.21 (1.69–2.88) **<0.0001**	1.75 (1.20–2.51) **0.0029**	1.51 (1.16–1.99) **0.0025**	1.34 (0.95–1.85) 0.0882
**Grade** **G1 vs. G2–G3**	1.13 (0.79–1.65) 0.4862		1.17 (0.80–1.71) 0.3768		0.76 (0.35–1.64) 0.5012	
**Stage** **I–II vs. III–IV**	2.20 (1.84–2.63) **<0.0001**	1.40 (1.08–1.82) **0.0088**	2.35 (1.80–3.10) **<0.0001**	1.38 (0.89–2.07) 0.1322	1.87 (1.41–2.48) **<0.0001**	1.30 (0.86–1.95) 0.1973
**Ki-67** **<25% vs. ≥25%**	0.96 (0.80–1.13) 0.6355		1.01 (0.73–1.38) 0.9410		0.91 (0.68–1.19) 0.4798	
**p63** **<25% vs. ≥25%**	0.83 (071–1.01) **0.0512**	0.82 (0.67–0.96) **0.0215**	0.90 (0.63–1.32) 0.6228		0.68 (0.47–0.98) 0.0371	
**TTF-1** **<25% vs. ≥25%**	0.98 (0.82–1.19) 0.1572		0.81 (0.59–1.10) 0.1807		1.42 (0.93–2.16) 0.0912	
**PD-L1** **0–1 vs. >1**	1.18 (0.90–1.56) 0.1975		1.54 (1.03–2.30) **0.0322**	1.46 (0.98–2.20) 0.0649	1.07 (0.70–1.63) 0.7082	

Abbreviations: HR—hazard ratio, CI—confidence interval, NSCLC—non-small cell lung cancer, AC—adenocarcinoma, SCC—squamous cell carcinoma; significance in bold.
